# Push-Out Bond Strength, Characterization, and Ion Release of Premixed and Powder-Liquid Bioceramic Sealers with or without Gutta-Percha

**DOI:** 10.1155/2021/6617930

**Published:** 2021-05-06

**Authors:** Cristina Retana-Lobo, Mario Tanomaru-Filho, Juliane Maria Guerreiro-Tanomaru, Marianella Benavides-García, Erick Hernández-Meza, Jessie Reyes-Carmona

**Affiliations:** ^1^Department of Restorative Sciences, University of Costa Rica, Montes de Oca, San José 11502, Costa Rica; ^2^Department of Restorative Dentistry, Universidade Estadual Paulista (UNESP), Araraquara, SP 14800-903, Brazil

## Abstract

**Objective:**

To evaluate the push-out bond strength of premixed and powder-liquid bioceramic sealers with or without gutta-percha (GP) cone.

**Materials and Methods:**

Radicular dentin samples were prepared from 80 single-rooted human teeth. After root canal preparation using ProTaper® and irrigation with NaOCl and EDTA, teeth were divided according to the root canal sealer (*n* = 20): AH Plus®, EndoSequence® BC Sealer™, ProRoot® Endo Sealer, and BioRoot™ RCS. Samples were randomly divided into two subgroups (*n* = 10): GP-S: root canal filling using the single-cone technique, or S: filling with only sealer. Specimens were kept at 37°C and 100% humidity in calcium-free PBS for 30 days. The push-out bond strength was measured in MPa. Fractured specimens were observed at 25x to evaluate the type of failure. pH and calcium ion release were measured at different experimental periods. Raman and SEM-EDAX analyses were performed for root canal sealers. Data were analysed using three-way analysis of variance (ANOVA) and post hoc Tukey test at a significance of *P* < 0.05.

**Results:**

Push-out bond strength was greater for samples obturated with only sealers (S) than samples obturated with the single-cone technique (GP-S) (*P* < 0.05). BioRoot™ RCS had greater bond strength than EndoSequence® BC Sealer™. Adhesive failures between cement and gutta-percha cone (87.5%) were predominant in the GP-S. Cohesive failures were predominant for S (80%). BioRoot™ RCS and ProRoot® ES presented higher alkalinization potential than the premixed sealer (EndoSequence® BC Sealer™). Powder-liquid bioceramic sealers (BioRoot™ RCS and ProRoot® ES) released the highest cumulative amount of calcium (28.46 mg/L and 20.05 mg/L).

**Conclusion:**

Push-out test without gutta-percha cone presents higher bond strength for bioceramic sealers. Powder-liquid calcium silicate-based sealers present greater bioactivity related to alkalinization potential and calcium ion release.

## 1. Introduction

Root canal obturation prevents infection related to either leakage or reinfection of the root canal system [1]. Root canal filling materials should adhere to dentin to achieve tridimensional sealing and improve the long-term success of endodontic treatment [2]. Endodontic sealers establish a connection between the dentin and the gutta-percha (GP) core and seal irregularities in root canals. Therefore, adhesive properties of the sealers are desired to obtain an adequate seal [3–6]. Dislodgement resistance or push-out bond strength is a parameter used to assess interfacial bonding between the materials and intraradicular dentin [1, 7, 8].

Endodontic sealers based on calcium silicate formulations have been developed due to the bioactive properties presented by mineral trioxide aggregate (MTA) [2, 4, 9]. The bioactivity of these cements can be attributed to their capacity to form carbonate apatite precipitates in the presence of phosphate-buffered saline (PBS) [10–14]. EndoSequence® BC Sealer™ (Brasseler USA, Savannah, USA) is a premixed bioceramic sealer. Its major components include tri- and dicalcium silicates, calcium phosphates, calcium hydroxide, and filler and thickening agents. Studies have shown that it has adequate characteristics such as flowability, dimensional stability, and bond strength [2, 6, 7, 15, 16].

Other bioactive sealers are available in a powder/liquid presentation, such as ProRoot® Endo Sealer (Dentsply Tulsa Dental Specialties, Ballaigues, Switzerland). The major components of the powder are tri- and dicalcium silicate, calcium sulphate, and bismuth oxide, and the liquid is a viscous aqueous solution of a water-soluble polymer [9]. It exhibits advantageous characteristics such as sealing ability, biocompatibility, and resistance to dislodgement [9, 17]. Furthermore, BioRoot™ RCS (Septodont, Saint-Maur-des-Fossés, France) is a mineral-based root canal sealer in a powder/liquid presentation. The powder composition mainly consists of tricalcium silicate, zirconium oxide, and povidone. The liquid part is an aqueous solution of calcium chloride with polycarboxylate [18]. BioRoot™ RCS presents biocompatibility and bioactivity and displays an adequate seal with dentin and GP [19]. Epoxy resin-based sealers such as AH Plus® (Dentsply DeTrey, Konstanz, Germany) is considered the gold standard for physicochemical properties [2, 4]. However, the main disadvantage of AH Plus® is the lack of bioactive properties [2].

The push-out bond test is widely used to assess the adhesive properties of different filling materials but with some limitations. It has been stated that it is not useful for thermoplastic materials, because of the plastic deformation of GP [8]. Therefore, root canal filling with a sealer without GP is one of the solutions suggested [8, 20, 21]. In spite of this, the deformation of the core material does not preclude the usefulness of the test to evaluate the dislodgement resistance of root-filling materials [8].

Thus, the objective of our study was to evaluate the push-out bond strength of premixed and powder-liquid bioceramic sealers with and without GP. The null hypothesis was that there is no difference in the bond strength between premixed and powder-liquid calcium silicate-based sealers with or without the use of a GP core.

## 2. Materials and Methods

The research protocol was approved by the Ethics Committee of Universidad de Costa Rica (VR-467-2018).

### 2.1. Sample Preparation

Radicular dentin samples were prepared from 80 extracted human teeth. The criteria for inclusion were single canal, straight root, and fully developed apices. The exclusion criteria were teeth with caries, resorptive defects, cracks, complex anatomy, and previous endodontic treatment. Specimens were cleaned mechanically to remove soft tissue and debris and stored in 0.1% thymol solution until use.

The crowns and the last 1 mm of the apical portion of the roots were removed using a water-cooled low-speed ISOMET diamond saw (Buehler, Lake Bluff, NY, USA) to standardize the teeth. The working length was calculated by subtracting 1 mm from the teeth length.

Root canals were prepared using a ProTaper System® (Dentsply Maillefer, Ballaigues, Switzerland) up to file F5 (50/05) at the working length and irrigated with 10 mL of 2.5% NaOCl throughout instrumentation. A standardized method of irrigation was performed using a 27-gauge Endo-Eze irrigation needle (Ultradent Products Inc., South Jordan, Utah, USA), placing the needle as deep as possible into the canal without binding and ejecting the irrigation solution gently. The final irrigation protocol to remove the smear layer was performed with 2 mL of 17% EDTA followed by 5 mL of 2.5% NaOCl [6]. Root canals were rinsed with distilled water and dried with paper points (Dentsply Maillefer, Ballaigues, Switzerland).

Samples were divided according to the root canal sealer (*n* = 20): Group 1: AH Plus®; Group 2: EndoSequence® BC Sealer™; Group 3: ProRoot® Endo Sealer; and Group 4: BioRoot™ RCS (Table 1). Samples were randomly divided into two subgroups (*n* = 10). In the GP-S subgroup, root canals were obturated to the working length using the single-cone technique with a F5 guttapercha cone (Dentsply Maillefer, Ballaigues, Switzerland). The sealers were manipulated according to the manufacturers' instructions and applied into the canals with an F5 paper point (Dentsply Maillefer, Ballaigues, Switzerland). The master cone was also lightly coated with the sealer and seated to working length. In the subgroup S, the root canals were filled with the sealers using a narrow diameter syringe with a 29-gauge NaviTip (Ultradent Products Inc., South Jordan, Utah, USA).

The teeth were stored at 37°C and 100% humidity in calcium-free PBS for 30 days. Each specimen was then sectioned perpendicular to the longitudinal axis of the root using an ISOMET diamond saw (Buehler, Lake Bluff, NY, USA). Three 1 ± 0.1 mm thick slices were prepared in the apical, middle, and coronal thirds [7]. The thickness of each slice was measured using a digital caliper.

### 2.2. Push-Out Bond Strength Evaluation

The root filling in each specimen was subjected to loading using a universal testing machine (H10KS, Tinius Olsen Testing Machine Company, Horsham, PA, USA). The loading speed was 1 mm/min. 0.5 mm–0.8 mm diameter cylindrical steel punch tips were used. The specimens were positioned with the apical aspect facing the punch tip to avoid any constriction interference due to the root canal taper. Push-out bond strength values in MPa were calculated as the force (N) of dislodgement divided by the surface area (A) of the bonded interface (mm^2^), using the standard equation (Equation (1)) for the frustum of a cone:

(1) $$\begin{array}{c} A=\pi \left(\text{R}1-\text{R}2\right)\sqrt{h^{2}+{\left(R1-R2\right)}^{2}}, \end{array}$$

where *π* is the constant 3.14, R1 is the larger radius, R2 is the smaller radius, and *h* is the thickness of the specimen.

After the push-out strength test, specimens were observed under a stereomicroscope at 25x to evaluate the types of failures. Different types of failure were observed: adhesive failure between cement/gutta-percha cone, adhesive failure between cement/dentin, cohesive failure in the material, and mixed failure.

### 2.3. Determination of pH and Calcium Ion Release

Specimens from all the experimental groups were placed separately in sterile vials and immersed in a calcium-free and magnesium-free phosphate-buffered saline (PBS) solution at 37°C for 1 month. The PBS solution was collected and replaced at 1, 3, 5, 10, 15, and 30 days to measure the pH and calcium ion release [21]. After collection of the solution, the pH was determined with a pH meter (Orion Star A 221, Waltham, MA, USA). Calcium ion release was measured using a Varian atomic absorption spectrophotometer (Spectra A220 Fast Sequential, Palo Alto, CA, USA). Data obtained were recorded and subjected to descriptive analysis.

### 2.4. Raman Analysis

Two specimens from each group were subjected to Raman analysis (*n* = 16) to evaluate the sealer chemical composition. Raman spectra were recorded using a Raman microspectrometer (ProRaman-L, Enwave Optronics Inc., Irvine, CA, USA). A 50x microscope objective (Leica Microsystems Inc., Buffalo Grove, IL, USA) was used, and the samples were excited using 45–50 mW of a 785 nm diode laser. The Raman signal was collected at a spectral interval of 200–1800 cm^−1^. The integration time was 40 s, and the spectral resolution was approximately 2 cm^−1^. All the reported spectra are the average of at least five measurements.

### 2.5. Scanning Electron Microscopy (SEM)

Two random specimens from each subgroup were selected for SEM observation (*n* = 16). Slices were prepared for SEM observation [22]. Specimens were dried at room temperature, mounted on an aluminium stub, placed in sealed glass vials with silica and then in a vacuum chamber, and sputter-coated with a 300 Å gold layer. The elemental composition of the sealers was analysed by energy-dispersive X-ray analysis (EDAX) with a scanning electron microscope (S-570, Hitachi, Tokyo, Japan) at 15 kV. Two evaluations were performed for each sample. Serial SEM photomicrographs at different magnifications between 40 and 5000x were taken to analyse the ultrastructure and the dentin-sealer interface.

### 2.6. Statistical Analyses

All statistical analyses were performed using Prism 9 GraphPad (GraphPad Software Inc., San Diego, CA, USA). Three-way analysis of variance (ANOVA) and the post hoc Tukey test were used (*P* < 0.05).

## 3. Results

### 3.1. Push-Out Bond Strength Evaluation

**Figure 1:**
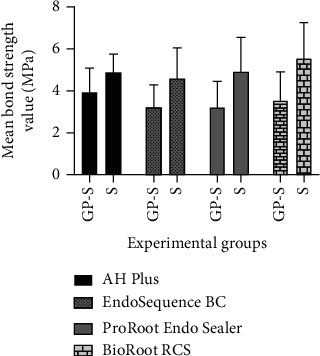
Push-out bond strength values (MPa) for the experimental sealers using the sealer only (S) and the GP core and sealer (GP-S).

The mean values of push-out strength (MPa) are shown in Table 2 and Figure 1. All S samples displayed a significantly greater resistance to displacement than the samples obturated with GP-S (*P* < 0.05). Bond strength was significantly greater in samples obturated with BioRoot™ RCS than with EndoSequence® BC Sealer™, regardless of the obturation technique (*P* < 0.05) (Table 2). The failure modes recorded were, mostly, adhesive between cement and gutta-percha cone (87.5%) in the GP-S. In the S subgroup, cohesive failures in the material prevailed (80%). In these samples, it was possible to observe part of the sealer attached to the radicular dentin.

### 3.2. pH and Calcium Ion Release

**Figure 2:**
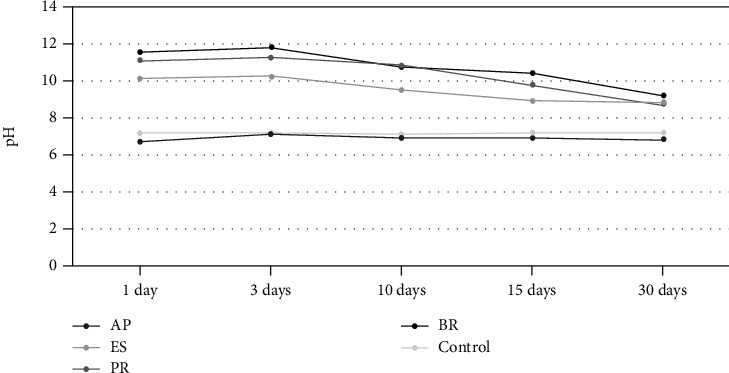
Alkalizing activity. pH profiles of the solutions. All the calcium silicate-based sealers showed a rapid initial rise, on day 3 followed by a decline on day 30. Control group and epoxy resin-based sealer remained near 7.2. AP: AH Plus™; ES: EndoSequence® BC Sealer™; PR: ProRoot® ES; BR: BioRoot™ RCS.

All calcium silicate-based sealers induced alkalinization of the PBS solution in a similar profile. The highest pH value was observed on day 3 and declined until day 30. The powder-liquid calcium silicate-based sealers presented higher alkalizing activity than the premixed sealer. AH Plus® showed a neutral pH (6.7–7.1) similar to the control group that remained stable at 7.2 throughout the experiment (Figure 2).

All the calcium silicate-based sealers showed the ability to release calcium ions. Powder-liquid sealers (BioRoot™ RCS and ProRoot® ES) released the highest cumulative amount of calcium (28.46 mg/L and 20.05 mg/L, respectively), and the levels remained high until the end of the experiment. The premixed sealer (EndoSequence® BC Sealer™) showed the maximum calcium release on days 1–3 (4.42 mg/L), which then decreased (2.42 mg/L). Epoxy resin-based AH Plus® showed a lower cumulative amount of calcium ions (7.34 mg/L). Trace amounts of calcium were detected in the control group (0.86–1.15 mg/L) (Table 3).

### 3.3. Composition and Structural Observation

**Figure 3:**
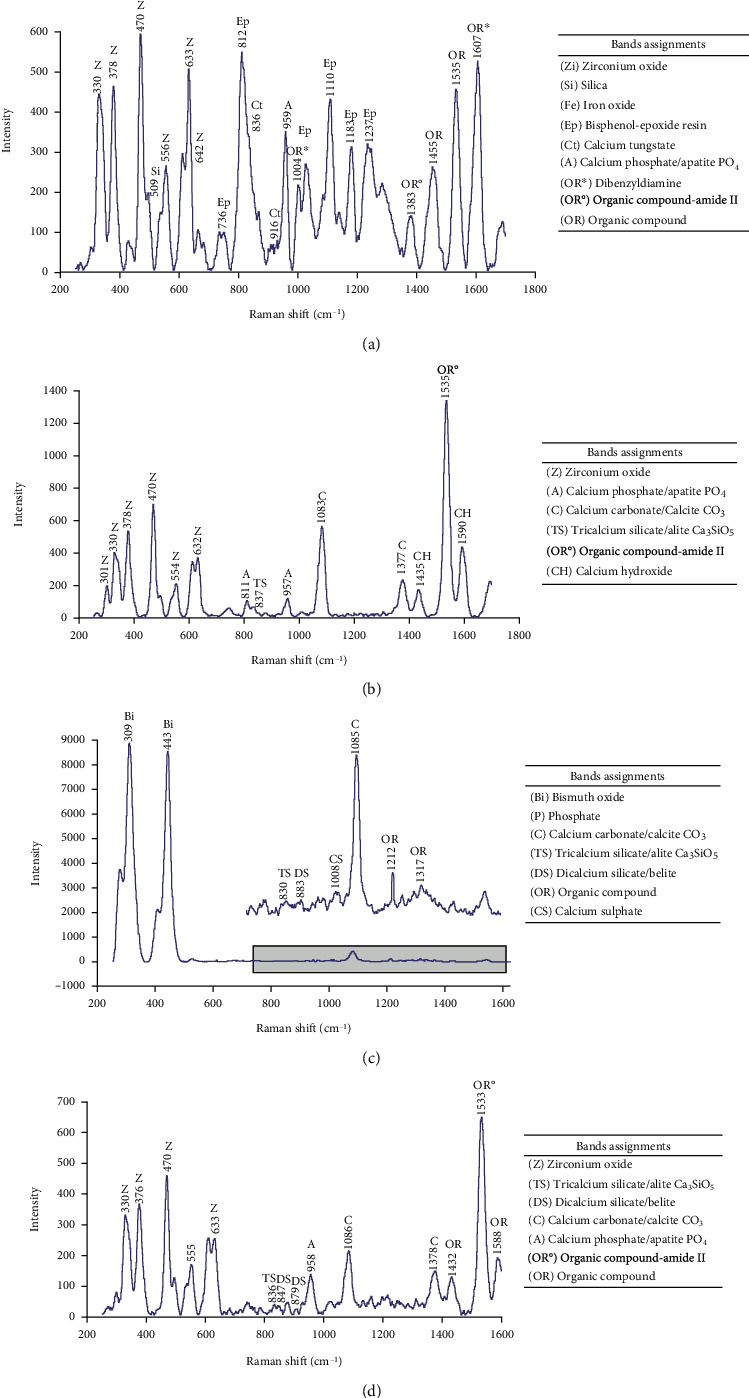
Average micro-Raman spectra: (a) Raman spectra obtained from AH Plus™; (b) EndoSequence® BC Sealer™; (c) ProRoot® ES, square area showed the amplified zone overlapped by the intensity of the bismuth oxide signals; (d) BioRoot™ RCS.

The Raman spectra obtained from all sealers were in agreement with the reported compositions (Figure 3). ProRoot® ES showed intense bands ascribable to bismuth oxide overlapping other compounds; therefore, amplification of the 700 cm^−1^ to 1600 cm^−1^ spectral interval was performed.

**Figure 4:**
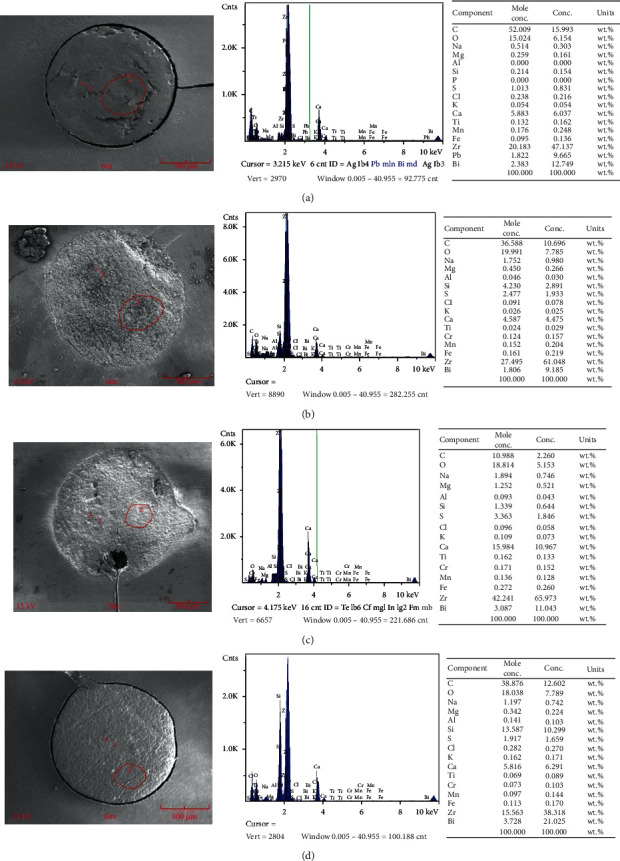
EDAX spectrum for (a) AH Plus™, (b) EndoSequence® BC Sealer™, (c) ProRoot® ES, and (d) BioRoot™ RCS.

The chemical compositions by EDAX of the experimental sealers were described in Figure 4. ProRoot® ES showed Ca and Zr at higher concentrations compared to the other sealers. BioRoot™ RCS detected Si in higher concentration.

**Figure 5:**
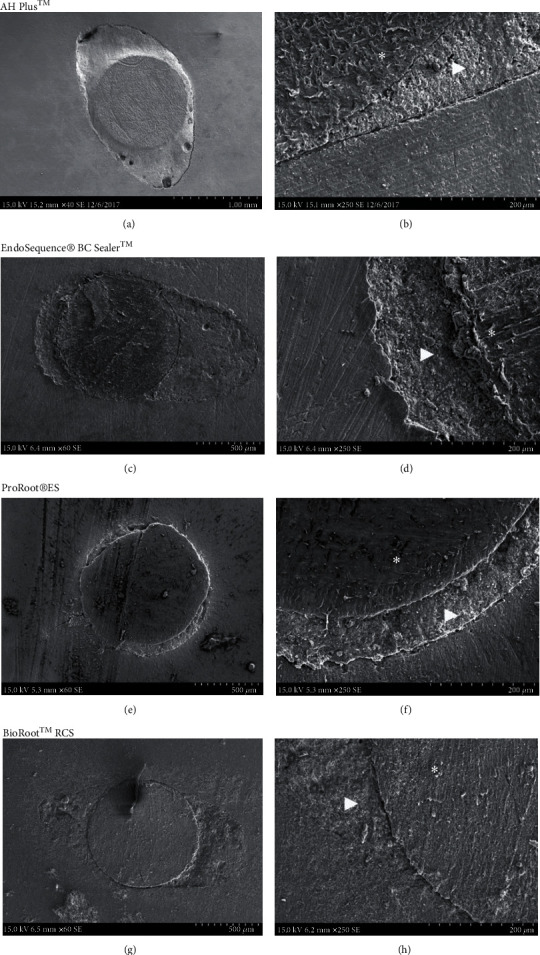
Representative photomicrograph of canals filled with calcium silicate-based cements and a GP core, single cone technique (a, c, e, g). Interface between the sealer (arrow), GP (∗), and dentin (b, d, f, h).

**Figure 6:**
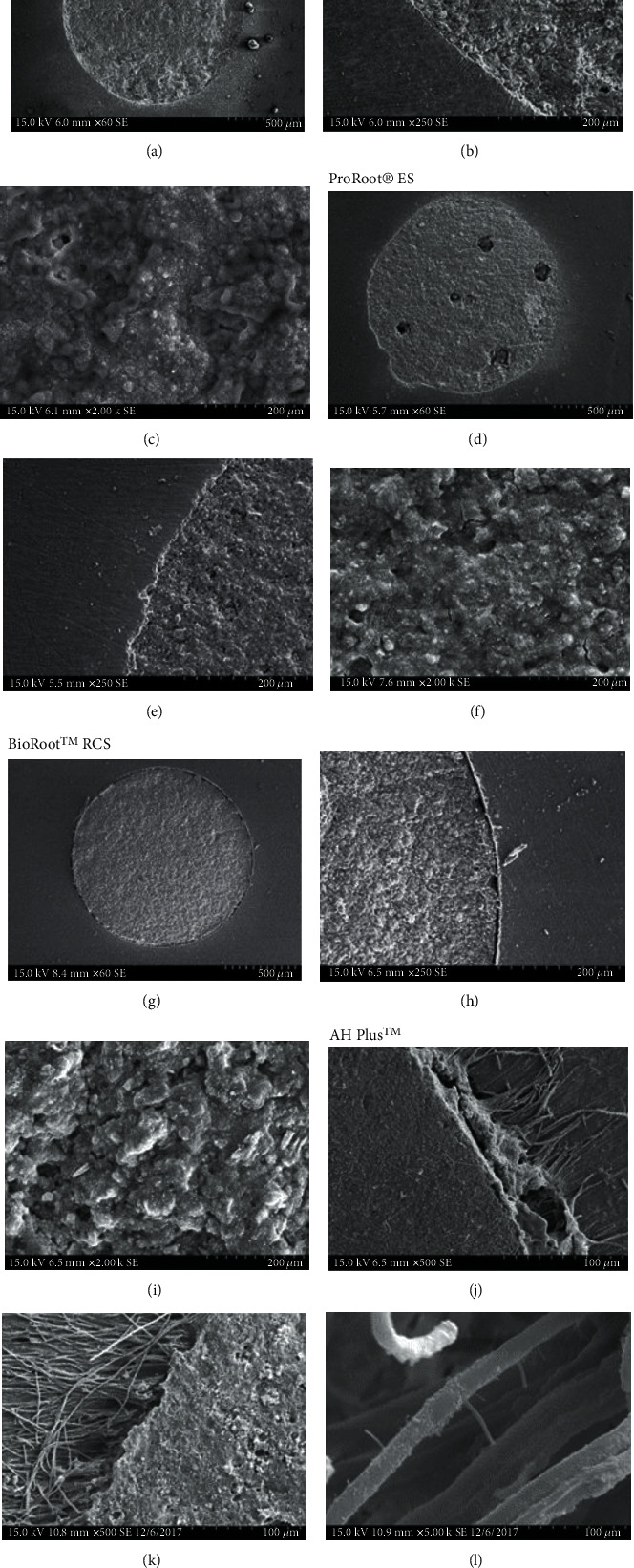
Representative photomicrographs of canals filled with only calcium silicate-based cements (a, d, g). Interface between the sealer and dentine (b, e, h) and photomicrograph of the sealers at high magnification (c, f, i). Interface between epoxy resin-based sealer AH Plus™ and dentin (j, k, l) and resin tags at higher magnification.

SEM evaluation showed dentin-sealer-GP interface. The sealer filled adequately the spaces between the GP and dentin (Figure 5).  Figure 6 showed the ultrastructure of the hardened sealers and adaptation to the dentinal walls of the samples obturated with only the sealers. All the groups showed adequate marginal adaptation to dentin and to GP (Figures 5 and 6). In AH Plus samples were observed some voids and marginal gaps at the dentin/cement interface. Bioceramic sealers showed a more homogeneous marginal adaptation and adequate adhesive sealing with dentin and GP.

## 4. Discussion

Bioceramic sealers promote biomineralization process, improving adhesion through mineral deposition in the root canal sealer-dentin interface [13, 23]. Our study was designed to evaluate the push-out bond strength of different calcium silicate-based sealers with and without GP as the core material. It was possible to observe significant differences in the bond strength between premixed and powder/liquid sealers. Moreover, our results showed that samples obturated with only sealers showed significantly greater bond strength than the samples obturated with the single cone technique. Therefore, these differences lead to rejection of the null hypothesis.

The push-out test is commonly used to assess the dislodgment resistance of different materials. It has been stated that this test does not entirely replicate the clinical performance of the root canal filling materials [1, 8, 24]; however, with the acknowledgment of its limitations, push-out tests are suitable [1, 8, 24].

In our study, the push-out test was performed after 30 days of PBS immersion of the specimens to simulate the clinical contact with body fluids and to evaluate the bond strength after the biomineralization process. Previous studies described that the potential bioactivity of calcium silicate-based materials is triggered when immersed in PBS increasing the dislodgement resistance [17, 25, 26]. Thus, Reyes-Carmona et al. [12] reported that bioactive cements release some components in PBS and trigger mineral deposition, which leads to the formation of an interfacial layer with tag-like structures in the intratubular dentin, suggesting that this biomineralization process could minimize leakage [12] and positively influence the push-out bond strength of the cements [13, 17]. This micromechanical interaction induced by calcium silicate cements improves the adhesion between materials and dentin [1, 13, 17, 27].

AH Plus®, an epoxy resin-based sealer, showed higher dislodgement resistance values when the single-cone obturation technique (GP-S) was performed. Our results are in agreement with several studies that demonstrated that AH Plus® had higher push-out bond strength values in comparison with calcium silicate-based sealers [1, 7, 28]. Donnermeyer et al. explained the higher resistance to dislodgement as a result of the covalent bonds between the epoxy resin and the amino groups of the collagen, resulting in a stronger link to dentin compared to the interaction of calcium silicates [1].

Although the use of only the sealer is not suggested clinically for root canal treatment, sealer-only experiments were included in this study to rank the bond strength exclusively related to the sealer itself. All the values for dislodgment resistance observed in our study were higher when the root canal was obturated with just the sealer. This is in concordance with Jainaen et al., who observed higher bond strengths when the filling was performed with the sealer alone than with GP and sealer [20]. Our results demonstrated that most adhesive failures occurred between the sealer and the GP cone, probably because sealers present stronger chemical and/or physical bonds to dentin than to the main GP core. Also, the plastic deformation of the GP core may negatively affect the push-out bond strength [5, 8].

BioRoot™ RCS showed higher bond strength values in comparison with the other experimental materials when the root canal was obturated with just the sealer. This calcium silicate-based sealer is able to nucleate carbonated apatite related to its ability to release calcium ions and maintain an alkaline environment [10, 18, 29]. Furthermore, Reyes-Carmona et al. reported the ability of bioactive materials to promote the biomineralization process in the interface between dentin and the cement, suggesting chemical bonding and, therefore, an improvement of the sealing ability [12]. Thus, we suggest that the higher bond strength shown by the BioRoot™ RCS (S) can be explained by the higher amounts of calcium ions released, suggesting greater biomineralization in the dentin-cement interface. The apatite precipitation is proportional to the concentration of available Ca^2+^ ions [12]. Therefore, it may be speculated that the samples obturated with only the sealer provided a greater amount of calcium ions, enhancing its biomineralization ability.

Our results showed that powder-liquid calcium silicate-based sealers presented higher calcium ion release and higher pH for all the experimental periods. BioRoot™ RCS and ProRoot® ES have shown higher leaching rates of Ca^2+^ and OH^−^ into PBS solutions [3, 18, 19]. The long-term release of calcium ions has been related to the tissue regeneration, improving the bioactivity and biocompatibility of the sealer [19]. Furthermore, alkaline pH promotes antibacterial effects and enhances the environment for mineral deposition [19].

EndoSequence® BC Sealer™ presented higher calcium and hydroxyl ions released up to day 10, after which they decreased. This fact may be explained by the final setting time of the material between 7 and 10 days [30]. These results are in contrast to those powder-liquid calcium silicate-based sealers, which presented higher values throughout the experiment. Siboni et al. stated that hydration processes continue after the final setting time, allowing CaP nucleation for up to 1 month [19]. AH Plus®, on the other hand, showed negligible amounts of calcium or hydroxyl ions, with values similar to the control group, in accordance with several studies that reported the absence of bioactive and alkalizing properties for this epoxy resin-based material [2, 18, 30].

Ultrastructural SEM examination allowed for observation of the dentin-sealer interface. Adaptation of the sealers to the dentinal walls was observed independently of the obturation technique.

Compositional EDAX and Raman analyses of the calcium silicate-based sealers showed the presence of di- and tricalcium silicates and radiopacifying agents such as calcium tungstate, zirconium oxide, bismuth oxide, calcium hydroxide, and calcium phosphate, in agreement with the manufacturer description for each material. Raman bands assigned to zirconium oxide were present in AH Plus®, EndoSequence® BC Sealer™, and BioRoot™ RCS. It has been reported that ZrO_2_ increases radiopacity and does not affect the setting of tricalcium silicate cements in controlled amounts [31]. Interestingly, ZrO_2_ has been related to a greater and longer release of calcium ions of di- and tricalcium silicate-based materials, maintaining long-term bioactivity [18, 32]. This could be associated with the finding that the BioRoot™ RCS group showed higher amounts of calcium ion release and presented higher values of push-out bond strength, especially when used alone, maintaining the bioactivity for long periods of time.

The continuous setting of calcium silicate-based sealers in the process of hydration and ion exchange with the medium could be related to the improvement of the bond strength and the stability of the sealing provided by the root canal filling in the long term [10, 12, 17, 33].

Suitable physicochemical properties have been described for all the bioceramic materials; however, additional research to assess the biological interaction of the calcium silicate-based sealers with the dentin matrix may provide information to improve clinical performance.

## 5. Conclusions

Push-out test without gutta-percha cone presents higher bond strength for bioceramic sealers. Powder-liquid calcium silicate-based sealers present greater bioactivity related to alkalinization potential and calcium ion release.

## Figures and Tables

**Table 1:** Materials, manufacturers, and composition of the tested sealers.

Sealer	Manufacturer	Lot	Composition
AH Plus™	Dentsply DeTrey GmbH (Konstanz, Germany)	1707000967	Paste A: diepoxide, calcium tungstate, zirconium oxide, aerosil, pigment (Fe oxide) Paste B: 1-adamantane amine; N,N-dibenzyl-5-oxa-nonandiamine-1,9; TCD-diamine; calcium tungstate; zirconium oxide; aerosil; silicone oil
EndoSequence® BC Sealer™	Brasseler USA dental (Savannah, GA, USA)	16001SP	Zirconium oxide, calcium silicates, calcium phosphate, calcium hydroxide, filler, and thickening agents
ProRoot® ES	Dentsply Tulsa Dental Specialties (Johnson City, TN, USA)	125606	Powder: tricalcium silicate, dicalcium silicate, calcium sulphate, bismuth oxide, and a small amount of tricalcium aluminate Liquid: viscous aqueous solution of a water-soluble polymer
BioRoot™ RCS	Septodont (Saint-Maur-des-Fossés, France)	B13365	Powder: tricalcium silicate, zirconium oxide, and povidone Liquid: aqueous solution of calcium chloride and polycarboxylate

**Table 2:** Mean bond strength value (MPa) and standard deviation recorded for each experimental group.

Bond strength (MPa)		
Groups	Mean	
AH Plus™	GP-S	3.959^a^ (1.12)
	S	4.897 (0.84)
EndoSequence® BC Sealer™	GP-S	3.223^b^ (1.06)
	S	4.579^b^ (1.45)
ProRoot® ES	GP-S	3.267 (1.19)
	S	4.953 (1.59)
BioRoot™ RCS	GP-S	3.522 (1.40)
	S	5.576^a^ (1.68)

Subgroups with different letters indicate statistically significant difference (*P* < 0.05).

**Table 3:** Calcium ion release (mg/L) and standard deviation of the different groups.

	Calcium ion release (mg/L)					
	1 day	3 days	10 days	15 days	30 days	Cumulative
AH Plus™	2.6 (0.3)	2.32 (0.6)	0.56 (0.01)	0.98 (0.2)	0.88 (0.2)	7.34
EndoSequence® BC Sealer™	4.22 (0.8)	4.42 (0.4)	2.40 (0.8)	1.87 (0.3)	2.42 (0.2)	15.33
ProRoot® ES	3.24 (0.7)	3.07 (0.3)	4.15 (1.3)	4.15 (1.3)	5.44 (1.2)	20.05
BioRoot™ RCS	3.82 (0.3)	7.76 (1.5)	4.13 (0.1)	6.30 (0.5)	6.45 (0.6)	28.46
Control	1.10 (0.06)	1.02 (0.04)	0.86 (0.1)	0.95 (0.03)	1.15 (0.3)	5.08

## Data Availability

Data availability, as stated for all theses from the University of Costa Rica, would be available and already archived in Repositorio Kerwa and the open access repository of our university.
